# COVID‐19 Persian Misinformation Detection on Instagram: A Comparative Analysis of Machine Learning and Deep Learning Methods

**DOI:** 10.1049/htl2.70036

**Published:** 2025-12-01

**Authors:** Mohammad Javad Shayegan, Elahe Moradi Pirbalouti, Mo Saraee

**Affiliations:** ^1^ Faculty of Engineering, Department of Computer Engineering University of Science and Culture Tehran Iran; ^2^ School of Science, Engineering and Environment University of Salford Salford UK

**Keywords:** CNN, COVID‐19, deep learning, LSTM, Persian misinformation detection

## Abstract

The proliferation of misinformation on social media platforms like Instagram poses a significant threat, eroding public trust and potentially endangering public health. While research in this area has grown considerably, a notable gap remains in addressing misinformation in languages other than English. This study bridges that gap by establishing a comprehensive performance benchmark for detecting misinformation on Instagram, focusing specifically on Persian‐language content related to COVID‐19. To support this research, we constructed a novel labelled dataset of 27,000 Persian comments collected from Instagram. We employed five established machine learning and deep learning approaches for misinformation analysis: XGBoost (with TF‐IDF embedding), LSTM (with GloVe embedding), CNN, KNN and BERT. To prevent overfitting, we conducted model training and validation, evaluating performance using confusion matrices. The results demonstrate that the LSTM model achieved the best classification performance, with an accuracy of 0.97, precision of 0.91, recall of 0.85 and an F1‐score of 0.85. This outcome establishes the first high‐performance baseline for deep learning methods on this unique Persian misinformation dataset. Furthermore, the training and validation curves for the LSTM approach indicate its effectiveness in mitigating overfitting.

## Graphical abstract

Despite extensive research on detecting misinformation in the context of COVID‐19, studies focusing on specific languages like Persian are limited. Particularly, there is a lack of labelled Persian datasets in this domain. The aim of this research is to propose a model for detecting misinformation, especially regarding COVID‐19. The highlights of this study are as follows:

• Constructed the first labelled dataset of Persian COVID‐19 misinformation on Instagram

• Developed a hybrid machine learning methodology for Persian misinformation detection

• Improved efficiency criteria for misinformation detection algorithms in Persian

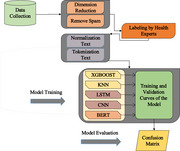

## 1 Introduction

The proliferation of COVID‐19 information on social media platforms in recent years has raised growing concerns about the physical and mental well‐being of individuals in society, highlighting the critical importance of this issue. Failure to identify misinformation can result in significant problems and costs for users. Misinformation on social media, whether in the form of text, images or videos, presents diverse challenges for analysis. Extracting features from each modality is difficult, emphasising the necessity of a large‐scale, comprehensive dataset for effective detection. The analysis of misinformation faces numerous challenges, including data volume, quality, domain complexity, interpretability, explainability, feature enrichment, model privacy and temporal modelling. Machine learning algorithms play a crucial role in data classification, modelling and analysis and are used in COVID‐related research such as screening, contact tracing and prediction. Deep learning models offer promising capabilities for detecting misinformation by accommodating complex interaction patterns and reflecting user preferences [1]. While extensive research has been conducted on analysing COVID‐19 misinformation, a significant gap remains in studies focusing on Persian‐language content, particularly on Instagram. Unofficial statistics indicate that Instagram is the most popular social media platform in Iran, with over 45 million users out of an approximate population of 88 million, despite being filtered by the government. Although COVID‐19 information is abundant on Instagram, there is currently no publicly available labelled Persian dataset for misinformation analysis. This complicates machine learning efforts, especially when working with Persian language content. To address these challenges, a dataset specifically tailored for analysing Persian COVID‐19 information on Instagram was collected for this research. Due to the absence of labelled data, manual labelling was conducted through interviews, consultations with health experts and references to reputable medical journals. This research aims to provide a comprehensive analysis of COVID‐19 misinformation on Instagram, with a specific focus on the Persian language. By leveraging deep learning methods, particularly those generalised from prior studies, this work seeks to contribute to understanding misinformation dynamics in the Persian context. Choudrie et al. [2] investigated misinformation classification using four machine learning algorithms and two deep learning models. Hayawi et al. [1] examined the training and validation curves for the XGBoost, long short‐term memory (LSTM) and bidirectional encoder representations (BERT) models. This research extends the models proposed in [1, 2] to the Persian language.

The contributions of this paper are summarised as follows:

- Construction of a labelled, specialised dataset of Persian COVID‐19 information from Instagram.
- Development of a methodology for analysing and detecting misinformation in the Persian language.
- Establishment of the first comprehensive benchmark comparing the performance of five established machine learning and deep learning architectures (XGBoost, KNN, CNN, LSTM and BERT) for Persian COVID‐19 misinformation detection.
- Demonstration of the comparative effectiveness of adapting existing deep learning techniques [1, 2] to establish a strong performance baseline in the resource‐scarce Persian language domain.

This study will contribute to the expanding body of research on COVID‐19 misinformation by offering insights into the specific challenges and opportunities associated with the Persian language domain on Instagram. The findings may inform the development of more effective strategies for mitigating the spread of misinformation and promoting public health during future outbreaks.

## 2 Related Work

### 2.1 Misinformation Analysis in English Language

Several studies have explored machine learning and deep learning techniques for analysing misinformation. Röchert et al. [3] developed a BERT‐based model for analysing misinformation on YouTube. Papadamou et al. [4] presented a misinformation video identification model with an accuracy of 79%, outperforming Random Forest (RF) and BERT. Choudrie et al. [2] investigated misinformation classification using various algorithms, including RF, SVM, decision tree (DT), Stochastic Gradient Descent (SGD), LSTM and CNN. Hayawi et al. [1] conducted a comparative evaluation of XGBoost, LSTM, and BERT models. Specifically focusing on COVID‐19 misinformation, Al‐Rakhami and Al‐Amri [5] performed binary classification by extracting features at both the tweet and the user level. Several studies have proposed deep learning approaches for handling unlabelled and imbalanced data. Islam et al. [6] recommended the use of NLP techniques to improve misinformation detection. Dong and Qian [7] proposed bidirectional recurrent neural networks (Bi‐RNNs) for misinformation detection with limited labelled data and large‐scale unlabelled data. Kumari et al. [8] explored sentiment label extraction with the BERT model, demonstrating its effectiveness compared to LSTM and multi‐channel CNN (MC‐CNN). Graham‐Kalio et al. [9] proposed and evaluated three models (support vector classifier [SVC], DT and logistic regression [LR]) for classification using 10‐fold cross‐validation. Alenezi and Alqenaei [10] addressed the issue of imbalanced datasets by applying a random sampling method. Several studies have explored the connection between sentiment analysis and misinformation detection. Kumari et al. [11] developed and evaluated multiple models for both sentiment analysis of the data and classification of fake information. Daradkeh [12] investigated the use of latent dirichlet allocation (LDA), a topic modelling technique, to identify dominant themes in misinformation. Didi et al. [13] suggested that combining TF‐IDF syntactic features with semantic features extracted using FastText and GloVe embeddings can improve classification performance. Sharma et al. [14] explored a BERT algorithm for topic modelling approaches based on semantic allocation and LDA. Du [15] investigated topic modelling using Latent Semantic Analysis (LSA) and the Biterm Topic Model (BTM). Safarnejad et al. [16] modelled information dissemination as a non‐homogeneous Poisson process signal. They employed content‐based features extracted using the language inquiry word count (LIWC) tool and word‐to‐vector conversion (word2vec). Cartwright et al. [17] investigated algorithms, including Posit's TensorFlow, and established text classification programmes like Libshorttext and Liblinear. Ravichandran and Keikhosrokiani [18] combined a deep neural network (DNN) with an adaptive neuro‐fuzzy inference system (ANFIS) to improve misinformation classification accuracy. Roy et al. [19] implemented algorithms with TF‐IDF embedding for feature extraction. Camelia et al. [20] developed machine learning models to detect fake news using a dataset of over 44,000 articles. Kuruva and Chiluka [21] applied deep learning models, including LSTM, to Twitter sentiment analysis on a dataset of 1.6 million tweets. İncir et al. [22] conducted monolingual and multilingual news classification and achieved the best results with DT and LR models. Chabukswar et al. [23] achieved the highest accuracy using a Bi‐LSTM model, combined with techniques like Word2Vec and GloVe. Purohit et al. [24] analysed sentiment in 143,000 news reports using RNNs, LSTMs, BERT and GPT. Alsuwat and Alsuwat [25] introduced a multimodal fake news detection framework that combines global, temporal, and spatial features using NLP techniques. Chen et al. [26] addressed two novel optimisation algorithms, KAdam‐EnGPT4LLM and GPT4ALL‐MediSentAly‐KAdam, to enhance efficiency and reduce training costs for patient feedback sentiment analysis. These two models led to faster convergence and more stable training, especially for medical questions and answers. Mahesh et al. [27] evaluated machine learning methods for breast cancer prediction and diagnosis. They also used the synthetic minority over‐sampling technique (SMOTE) to handle imbalanced data. The majority‐voting method, which was based on the top three classifiers (LR, SVM and classification and regression tree [CART]), provided the highest accuracy of 99%. Omar et al. [28] compiled a standard Arabic dataset from multiple platforms that was used to detect hate speech and abusive content. To validate the dataset's effectiveness, both machine learning and deep learning algorithms were used. The RNN achieved better performance with an accuracy of 98%. Khairy et al. [29] analysed 27 studies to review the findings of prior research regarding the detection of cyberbullying and offensive content in Arabic content on online social networks. Their goal was to help future researchers in developing effective and realistic automated detection systems. Omar and Abd El‐Hafeez [30] presented a comparative study on quantum computing and machine learning for two Arabic document classification datasets. In the first dataset, both approaches achieved high accuracy in sentiment analysis. In the second dataset, processing time was significantly reduced compared to the first. These results demonstrated the effectiveness of both models in accurately predicting sentiment. In order to build an efficient associative classifier, Farghaly et al. [31] combined association rule mining with SVM to reduce the number of extracted classification rules and improve prediction accuracy. The results showed that the model outperformed other classification approaches. Mostafa et al. [32] presented an approach to predicting hepatocellular carcinoma (HCC), comparing the performance of DT, Naive Bayes, KNN and SVM models both before and after applying feature reduction methods. The results showed that the reduced feature set outperformed the original dataset in terms of prediction accuracy and execution time.

### 2.2 Misinformation Analysis in Persian Language

As Table 1 shows, a comparison of related work is presented. Despite the extensive research conducted in the field of misinformation, significantly less attention has been paid to Persian misinformation. Comprehensive evaluations of methods such as XGBoost, LSTM, CNN, BERT and KNN are rare, as is the examination of their training and validation curves. Another challenge in analysing Persian COVID misinformation is the limited availability of standardised datasets and labelled data, particularly on the social media platform Instagram.

**TABLE 1:** Comparison of related work.

Reference	Approach	Method	Year
[6]	Review study	**Deep learning models**	2020
[2]	Comparative evaluation	**SVM, DT, RF, SGD, LSTM, CNN**	2021
[1]	Comparative evaluation	**XGBoost, LSTM, BERT**	2022
[3]	Ensemble models	**BERT, SVM, LR, LSTM, CNN**	2021
[5]	Group learning	**SVM, RF, KNN, DT, naive bayes**	2020
[7]	Bi‐RNN	**Supervised Bi‐RNN, unsupervised Bi‐RNN**	2022
[9]	Text classification	**DT, LR, SVC**	2021
[10]	Ensemble models	**LSTM, CNN, KNN**	2021
[12]	Sentiment analysis	**DT, SVM, Naive Bayes**	2022
[13]	Sentiment analysis	**TF‐IDF, Word2Vec, GloVe, FastText**	2022
[14]	Text classification	**BERT, RF, SVM, DT, KNN, Naive Bayes**	2022
[15]	LDA modelling	**Double Layered Neural Network**	2021
[17]	Integrated model	**Posit's TensorFlow, RF**	2022
[18]	Ensemble models	**ANFIS—DNN**	2023
[19]	Automatic analysis	**RF, LR, KNN, LSTM**	2023
[33]	Ensemble models	**XLM‐RoBERTa and CNN**	2022
[34]	Data polarisation	**LDA**	2021
[35]	Clustering	**SOM, K‐means**	2022
[36]	Ensemble models	**CNN, XGBoost, PSO**	2022
[37]	3D structure	**CNN**	2021
[20]	Feature classification	**LSTM**	2024
[21]	Sentiment analysis	**RNN, Bi‐RNN, LSTM**	2024
[22]	Multilingual data	**RF, LR, DT, KNN**	2024
[38]	Hybrid models	**LSTM‐CNN, BERT**	2024
[23]	Comparative evaluation	**RF, SVM, Bi‐LSTM**	2025
[24]	NLP and transformer	**RNN, LSTM, BERT, GPT**	2025
[25]	Multimodal detection	**Bi‐LSTM**	2025
[30]	Comparative evaluation	**Quantum computing, RF**	2023

## 3 Research Method

**FIGURE 1:**
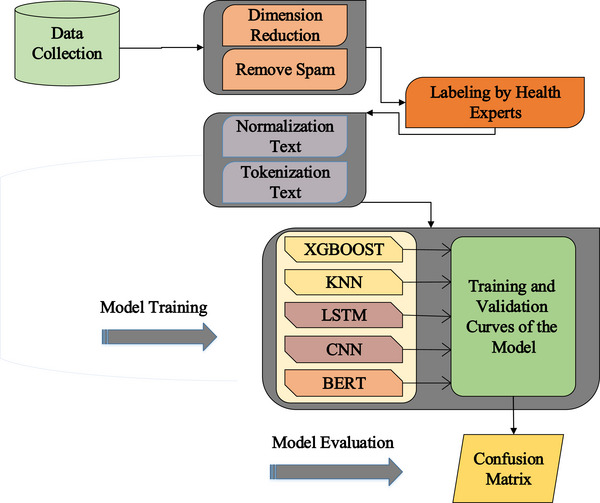
General research method.

Figure 1 shows the general steps of the reasearch method.

### 3.1 Data Collection

Due to the absence of a standard Persian dataset for COVID‐19 misinformation analysis on Instagram, data was collected directly from the platform between 5 September, 2022 and 11 December, 2022 [39]. A significant portion of this data was sourced from social media pages managed by healthcare organisations. Hashtag‐based searches were conducted to identify relevant content, including the following: ‘corona_china’, ‘corona_iran’, ‘minister_of_health’, ‘health_protocols’, ‘alcohol’, ‘mask’, ‘vaccine’, ‘astrazeneca’, ‘sinopharm’, ‘sputnik’, ‘barakat_vaccine’, ‘Pfizer’, ‘fakhra’, ‘omicron_in_children’, ‘buy_vaccine’, ‘covid_vaccine_national_demand’, ‘we_will_defeat_corona’, ‘we_make_vaccine’, ‘mandatory_vaccine’, ‘corona_phobia’, ‘take_corona_seriously’, ‘stay_at_home’ and ‘quarantine’.

An optimised data collection script was developed to extract specific features from Instagram. Selenium, a powerful web scraping tool, was employed because it is capable of handling dynamic web pages requiring user interaction. Python, along with the Selenium, NumPy, Pandas, Arabic‐Reshaper, Traceback and Datetime libraries, was used to automate the dynamic extraction process.

### 3.2 Data Labelling

Supervised learning models require labelled data for training. Due to the lack of a pre‐existing labelled dataset for COVID‐19 misinformation in Persian, the data labelling process was conducted manually by a group of healthcare professionals. This team comprised a medical doctor, a public health expert, and a virologist.

To enhance accuracy, validity and reduce labelling errors, additional interviews and consultations were conducted with two other healthcare experts. To ensure the accuracy of the data labels, thirty research papers published in esteemed medical journals on COVID‐related topics were used for verification.

**FIGURE 2:**
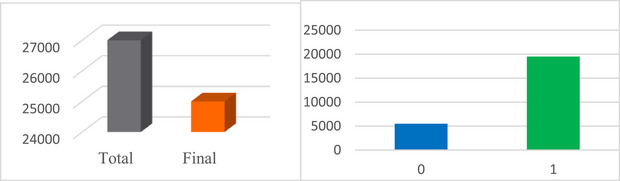
Labelling of the collected data.

These studies encompassed a broad range of topics, including healthcare, control measures, treatment strategies, self‐care, self‐medication practices, the potential link between COVID and other diseases, challenges in laboratory diagnosis, the role of quarantine in epidemic control, COVID‐19 in both children and adults, mental health considerations, supplements, the impact of nutrition, traditional medicine practices, psychological factors and COVID vaccine acceptance strategies. Figure 2 illustrates the labelling process, where data points deemed accurate were assigned a label of ‘1’ and those deemed inaccurate received a label of ‘0’. All comments were manually reviewed to ensure content accuracy. The data labeling process adhered to methodologies employed in previous studies [6, 40], as outlined below.

Data are categorised into three conceptual segments: proven (positive), unproven (unrealistic) and neutral.

- Data with unproven content are labelled as 0, and data with proven content are labelled as 1.
- Neutral data, including general and promotional comments, do not contain misinformation and are labelled as 1.
- Humorous and sarcastic comments that do not contain incorrect content are labelled as 1.
- Comments that contain information that is almost certainly wrong or contradict official guidelines are labelled 0.
- Questions that do not convey misinformation are labelled as 1.

### 3.3 Text Normalisation and Sentence Tokenisation

The text normalisation phase focuses on cleaning the text data and standardising characters. This step aims to replace non‐standard characters within the input text. The Hazm package, a Python library for natural language processing on Persian text, is employed to achieve word correction and normalisation within the text. The sentence tokenisation phase utilises a tokeniser tool to perform sentence detection on the input text.

### 3.4 Modelling

**FIGURE 3:**
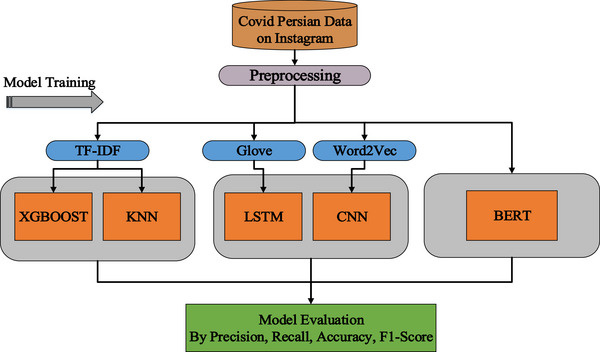
Components of the proposed model.

Figure 3 shows the components of the proposed model.

This methodology is designed to achieve the optimal balance between predictive accuracy, computational efficiency, and the ability to comprehend the subtleties of the Persian language in social media content, specifically on Instagram, for the detection of Persian COVID‐19 misinformation. The comparison of the three main model categories allows for a robust, multi‐faceted justification for the final model selection.

- **Traditional machine learning approach (ML baselines)**: We utilised TF‐IDF for text feature representation. This method extracts the most relevant features of the Persian Instagram content by statistically weighting words and helps us evaluate the effectiveness of simpler algorithms in detecting misinformation based solely on the statistical importance of words.
  - **Feature engineering (TF‐IDF)**: This technique extracts the most relevant features from Persian Instagram content by assigning statistical weights to words. It also enables assessment of simple algorithms’ performance in detecting misinformation based on word importance alone.
  - **XGBoost**: This ensemble model leverages the gradient boosting method. Its parallel architecture ensures high execution speed and reliable training, even with large datasets. Additionally, regularisation techniques effectively control overfitting and prevent excessive learning of random data noise.
  - **KNN**: KNN, a lazy learning model, is used to evaluate performance based on vector proximity within the TF‐IDF feature space. This simple model assesses the ability to detect new misinformation posts by measuring content similarity to previously identified posts.
- **Deep learning approach**
  These models were employed for the automatic extraction of semantic features:
  - **GloVe**: We generated or loaded GloVe embeddings using the Gensim or Keras libraries to create nuanced semantic representations of words in vector space.
  - **LSTM**: LSTM networks are highly effective for processing Persian text because they capture long‐term dependencies and sequential structures, crucial for handling the syntactic complexities of lengthy Persian sentences. These networks can recognise how keywords at the beginning of a sentence connect to the verb or final claim at the end. The model architecture includes a GloVe embedding layer, multiple LSTM layers and a Softmax dense layer for classification. Training utilised optimisation algorithms such as Adam or RMSprop.
  - **CNN**: Building on previous studies [36, 37] that explored text analysis and improved CNNs for spotting fake news. this model automatically discovers and leverages important features, resulting in better pattern recognition. It was applied for the automatic identification of local patterns and recurring key phrases (such as ‘miracle cure’ or ‘quick and definitive cure’) within short Instagram texts, yielding superior results in identifying these pattern types. CNNs can rapidly detect phrase patterns anywhere in the text, regardless of their position in the sentence.
- **Transformer Learning Approach**
  - **BERT**: To evaluate the maximum achievable accuracy, the ParsBERT model was employed. Based on the transformer architecture, this model leverages extensive pre‐trained linguistic knowledge. BERT can comprehend multi‐faceted contexts, such as understanding the meaning of a word in a humorous text, far better than traditional deep learning models. Initially, BERT was trained on general COVID‐19 data. Subsequently, a fine‐tuning process using the proprietary Instagram COVID‐19 dataset allowed BERT to adapt its general linguistic knowledge, focusing on misinformation patterns specific to this domain (health) and platform (Instagram).

**FIGURE 4:**
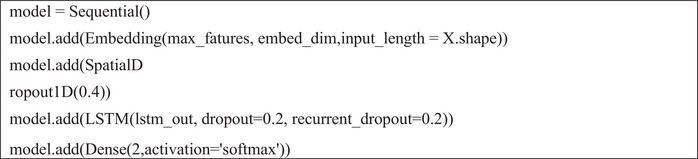
Implementing the LSTM model.

**FIGURE 5:**
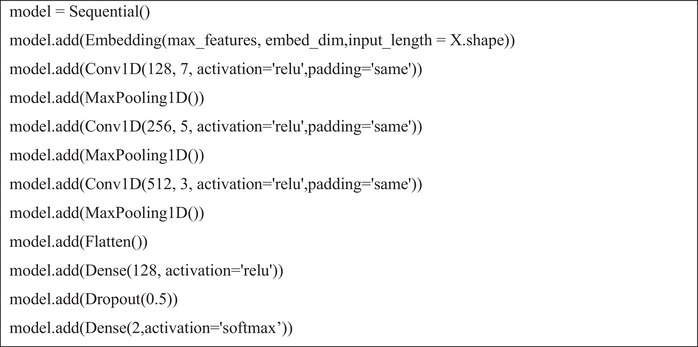
Implementing the CNN model.

**FIGURE 6:**
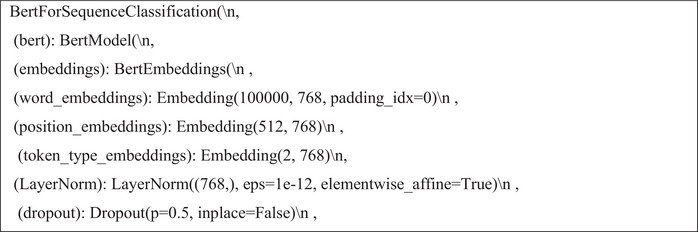
Implementing the BERT model.

After completing these steps, the LSTM model architecture was defined by specifying two key hyperparameters: embed_dim and lstm_out. The model was compiled using the Adam optimiser and categorical cross‐entropy loss, with softmax as the activation function. Figures 4, 5 and 6 illustrate the implementation of the LSTM, CNN and BERT models.

**FIGURE 7:**
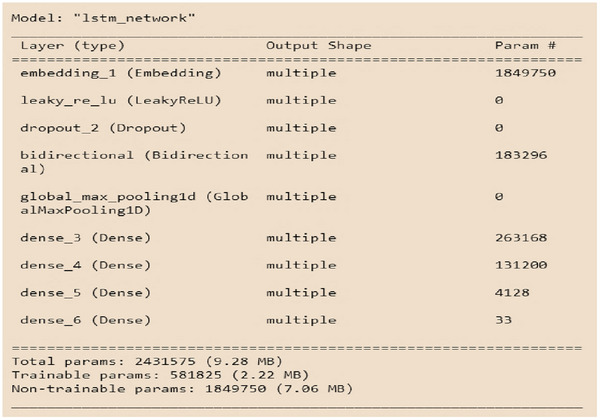
Implementing the **LSTM model**.

**FIGURE 8:**
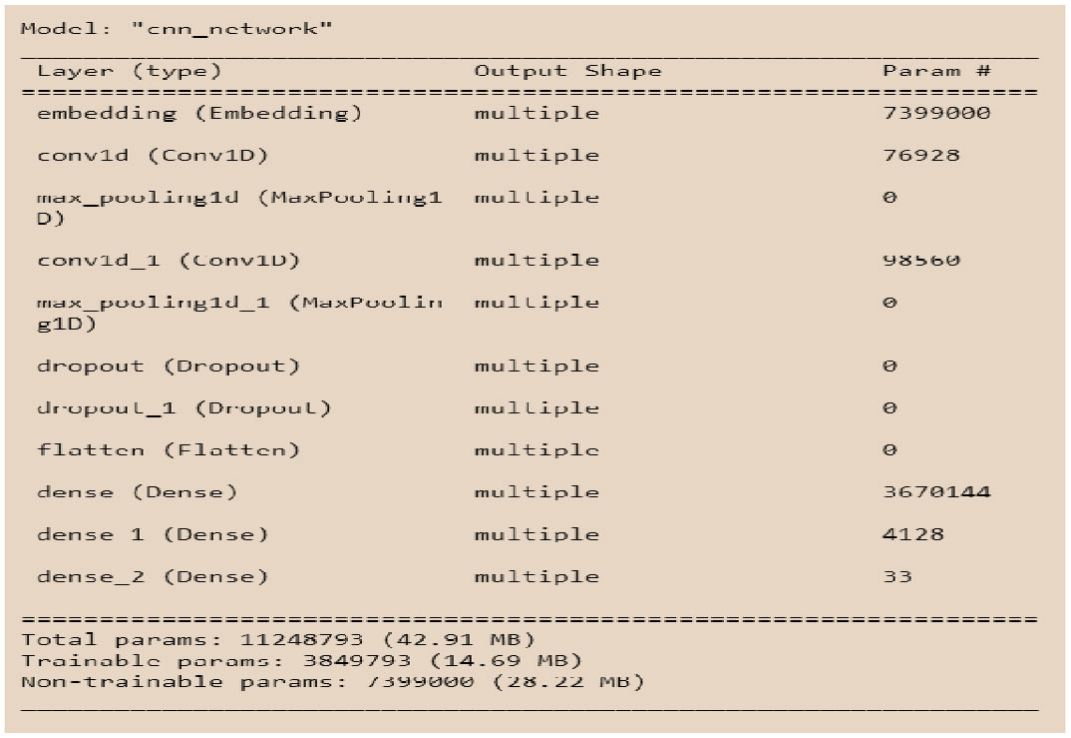
Implementing the **CNN model**.

In this study, five different learning models were investigated for training a detection model. Due to the similarity of the problem to sequence‐to‐sequence prediction, the LSTM model was selected as the primary choice. Figures 7 and 8 respectively show the architectural details of the LSTM and CNN models. The results indicate that the LSTM architecture achieved the highest classification accuracy. Furthermore, the F1‐score results also confirmed the superior performance of the LSTM. Regarding execution time, the models recorded 966 s for LSTM, 456 s for CNN, and less than 10 s for XGBoost.

Imbalanced data refers to datasets where the target class has a highly uneven distribution of observations, meaning one class contains a very high count of observations and another has a very low one. Accuracy is the total number of correct predictions made by a classifier divided by the total number of predictions. While this metric may be sufficient for a balanced class, it is not ideal for an imbalanced class problem. Other metrics, such as precision (which measures the accuracy of the classifier's positive predictions) and recall (which measures the classifier's ability to identify a class), are also important. For an imbalanced dataset, the F1‐score is a more suitable metric. If the classifier predicts the minority class but the prediction is wrong, and false positives (FPs) are high, precision will be low, which will also cause the F1‐score to decrease. Similarly, if the classifier has low recall, meaning a large number of the minority class are incorrectly classified as negative, the F1‐score will decrease.

With a high precision of 0.91, the model acted with great caution in detecting misinformation, resulting in a low number of FPs. In other words, it rarely misclassifies correct information as misinformation. However, due to the lower recall, the model had a higher number of false negatives (FNs), contributing to a lower F1‐score of 0.85. This means the model failed to identify a significant number of actual misinformation instances, mistakenly classifying them as correct. The main reason for this trade‐off is that the LSTM model performed better in terms of precision, accurately identifying which instances were misinformation, but was less effective in terms of recall, failing to find all of the misinformation. Such a trade‐off is common when data is imbalanced (e.g., when the amount of misinformation is very small relative to the total data or when the model is optimised to reduce FPs).

### 3.5 Scalability Analysis

Deep learning architectures like LSTM, BERT and CNN are generally more scalable for detecting misinformation in Persian COVID‐19 posts on Instagram than KNN and XGBoost. The scalability of each approach depends on its architectural complexity, computational requirements and its ability to handle a continuously growing dataset.

### 3.6 Highly Scalable Approaches

BERT is highly scalable due to its pre‐trained nature and transformer architecture. It can process massive amounts of text data efficiently and capture the complex context of Persian text. Once a BERT model is fine‐tuned on a specific task like misinformation detection, it can be deployed to classify new posts in real‐time. The main challenge is the high computational cost of the initial training, but inference (classification of new data) is relatively fast and can be scaled horizontally. CNNs are well‐suited for text classification and are highly scalable for this task. They can extract spatial features from text, which, in numerical form, allows them to process large datasets efficiently. Their parallelisable nature makes them ideal for deployment in distributed systems, handling the high throughput of Instagram posts. CNNs also benefit from pre‐trained word embeddings, which can improve performance and reduce training time. LSTMs are effective at capturing long‐term dependencies in sequential data like text. They are more scalable than traditional RNNs because they can handle longer sequences without suffering from vanishing gradients. While training an LSTM can be computationally intensive, its ability to process variable‐length text makes it a good fit for social media content. However, they are less efficient for parallel processing compared to CNNs or BERT, a factor that can limit their scalability in high‐volume scenarios.

### 3.7 Less Scalable Approaches

XGBoost is a powerful and efficient gradient boosting algorithm for tabular data. While it can be used for text classification by first converting text into features (like TF‐IDF), it is generally less scalable for handling unstructured text data like Instagram posts than deep learning models. As the dataset of posts and their corresponding features grows, the memory and computational requirements for building and updating the trees become a bottleneck. KNN's scalability is severely limited for large datasets. To classify a new post, KNN must compare it to every single post in the existing training dataset to find its ‘k’ nearest neighbours. As the number of Instagram posts increases, this comparison process becomes computationally prohibitive and very slow, making it impractical for real‐time or large‐scale applications. It's often used for smaller, proof‐of‐concept tasks rather than production‐level systems.

## 4 Results

The models were deployed on the Google Colab platform, using 12.7 GB of RAM, 15 GB of GPU RAM, and 78.2 GB of disk capacity. For implementation, we used the scikit‐learn library for XGBoost and KNN models, the TensorFlow library for creating CNN and LSTM models, and the Transformers library for loading and utiliding the BERT model. The effectiveness of the employed models was evaluated using standard metrics: accuracy, precision, recall and F1‐score.The presentation of utilided parameters (hyperparameters) for various algorithms is vital to ensure reproducibility and transparency. Table 2 presents a set of model‐specific parameters that directly influence training and model performance.

**TABLE 2:** Model‐specific parameters.

Model	Hyperparameter	Value used	Description/notes
**LSTM**	Optimiser	Adam (learning rate: 1e−3)	Used for updating model weights.
	Batch size	64	Number of samples per gradient update.
	Embedding dimension	300	Used pre‐trained Persian FastText vectors.
	LSTM units	128	Number of recurrent cells in the LSTM layer.
**BERT (fine‐tuning)**	Base model	ParsBERT	Specify the pre‐trained Farsi model used.
	Learning rate	2e−5	Crucial low rate for fine‐tuning.
	Max sequence length	128	Set based on 95th percentile of post length.
	Epochs	4	Fixed number of training passes.
**CNN**	Filter sizes	3, 4, 5	Kernel window sizes for convolution.
	Number of filters	100 per size	Total of 300 filters (3 × 100).
	Activation function	ReLU	Applied after the convolution layer.
**XGBOOST**	Feature engineering	TF‐IDF (max features: 5000)	Specify the text representation used.
	n_estimators	150	Number of boosting rounds (trees).
	Max depth	5	Maximum tree depth.
	Learning rate	0.1	Step size shrinkage.
**KNN**	Feature engineering	Word2Vec (dimension 100)	Specify the text representation used.
	K (n_neighbors)	9	Optimal number of nearest neighbours found via grid search.
	Distance metric	Cosine similarity	Metric used to calculate the distance between samples.

**FIGURE 9:**
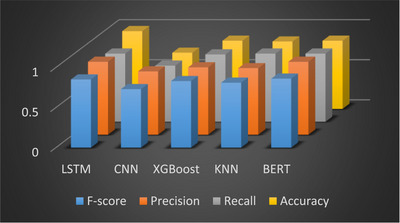
Analysis of false information based on evaluation criteria.

The results revealed that the LSTM model offers the optimal performance profile for this task, as demonstrably supported by the training history presented in Figure 9. LSTM achieved the highest accuracy of 0.97, a finding that highlights the particular efficacy of the LSTM approach for analysing Persian misinformation. Both the BERT and LSTM models demonstrated superior and comparable performance in terms of recall, each achieving a score of 0.85, and attained the highest precision with a score of 0.91. While both LSTM and BERT attained the maximum accuracy, the LSTM model achieved the highest F1‐score of 0.85 (as noted in the Conclusion) and demonstrated a superior balance of precision and recall compared to all other tested architectures. Additionally, the LSTM model exhibited faster training times than the CNN model, and critically, faster times than the computationally heavier BERT model. Considering the time‐sensitive nature of misinformation dissemination, where false information often spreads more rapidly than accurate information, the superior F1‐score coupled with the faster training speed of LSTM makes it an optimal choice for real‐time detection in resource‐constrained environments.

### 4.1 Addressing Performance in a High‐Stakes Context (Recall and FNs)

We acknowledge the critical point regarding the model's performance in a public health domain, where the minimisation of FNs (i.e., failing to detect harmful misinformation) is paramount. The achieved recall of, while representing the best performance among the tested architectures, still indicates a non‐trivial rate of FNs, which is a clear limitation for a high‐stakes detection system.

However, it is essential to consider the precision‐recall trade‐off in the context of social media content moderation. Our model's high precision of 0.91 ensures that when a post is flagged as misinformation, it is highly likely to be genuinely false. This high precision is crucial for maintaining platform credibility and user trust, minimising the risk of FPs, which could lead to the erroneous censorship of legitimate or satirical public health content. Therefore, the F1‐score of 0.85 represents the best initial balance achievable on this novel Persian dataset using established architectures. For future deployment, optimising the model to bias classification toward higher recall is the essential next step. While both LSTM and BERT models achieved high precision, LSTM showed a greater capability in identifying and extracting key features of misinformation. Notably, LSTM's training speed is faster than that of CNN, which is a significant operational advantage. Given that misinformation often spreads faster than accurate information, LSTM's high training speed makes it an ideal choice for real‐time detection. This study indicates that the LSTM model not only significantly outperforms architectures like CNN in terms of precision and training speed, but also exhibits very strong performance in accuracy compared to BERT. These findings introduce LSTM as an efficient tool for real‐time detection of misinformation in Persian social media content, taking into account the existing linguistic challenges. In the proposed approach, 80% of the total data is used for training the model to learn and extract patterns for misinformation detection, while the remaining 20% serves as the test set to evaluate final, real‐world model performance and ensure generalisation ability. Table 3 provides a comparative analysis of this research with previous studies on the topic.

**TABLE 3:** Comparison of previous studies.

Ref	Method	Best performance	Language	Dataset (N)	Best results			
					F1‐score	Precision	Recall	Accuracy
Röchert et al. [3]	BERT	BERT	English	10,400	0.97	0.97	0.96	—
Al‐Rakhami and Al‐Amri [5]	Ensemble model	SVM+RF	English	409,000	0.95	0.95	0.95	0.96
Hayawi et al. [1]	XGBoost, LSTM, BERT	BERT	English	15,073	0.98	0.97	0.98	0.98
Ghayoomi and Mousavian [33]	XLM‐RoBERTa, CNN	XLM‐RoBERTa, CNN	Bilingual	3,721	0.96	0.94	0.99	_
Mottaghi et al. [36]	Ensemble model	Bi_GRUCapsule+XGBoost+PSO	Persian	42,000	0.96	0.96	0.95	0.95
Mottaghi et al. [37]	3D_TCNN	3D_TCNN	Persian	42,000	0.93	0.94	0.93	0.94
Kar [40]	SVM, Naïve Bayes, RNN, CNN	SVM	English	149	—	—	—	0.95
Ghayoomi [41]	SVM, LR, RF	RF	Persian	122	0.56	—	—	0.57
Proposed method	LSTM, CNN, XGBoost, KNN, BERT	LSTM	Persian	27,000	0.85	0.91	0.85	0.97

### 4.2 Training and Validation

Training and validation were performed using precision, accuracy, recall and loss curves to assess potential overfitting. These curves provide an analytical approach for evaluating model performance during training.

**FIGURE 10:**
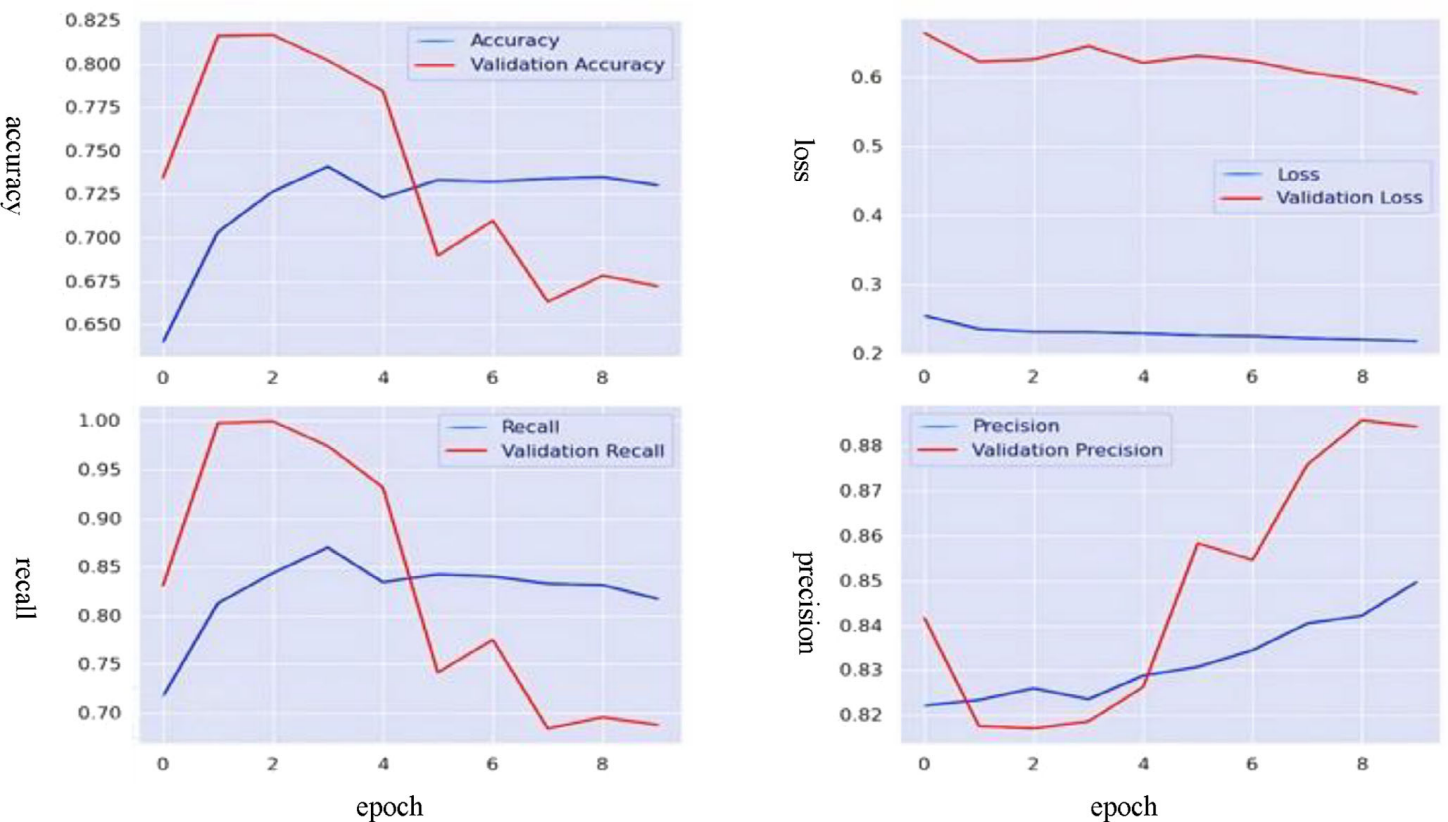
Training and validation curves of the **CNN model**.

Figure 10 illustrates the training and validation metrics of the proposed CNN approach over eight epochs. The maximum precision, accuracy and recall achieved were 0.75, 0.73 and 0.85, respectively, with a minimum validation loss of 0.25. However, the training and validation curves for accuracy and recall suggest a concerning decline in values as epochs increase, indicating reduced ability to correctly classify samples and identify true positives. Furthermore, the training and validation loss curves demonstrate overfitting. Loss, which measures the error between predicted and actual values, is minimised during model training to improve prediction accuracy. Validation loss, calculated using validation data, helps assess the model's generalisability. Based on these curves, the proposed CNN model failed to generalise effectively and was prone to overfitting.

**FIGURE 11:**
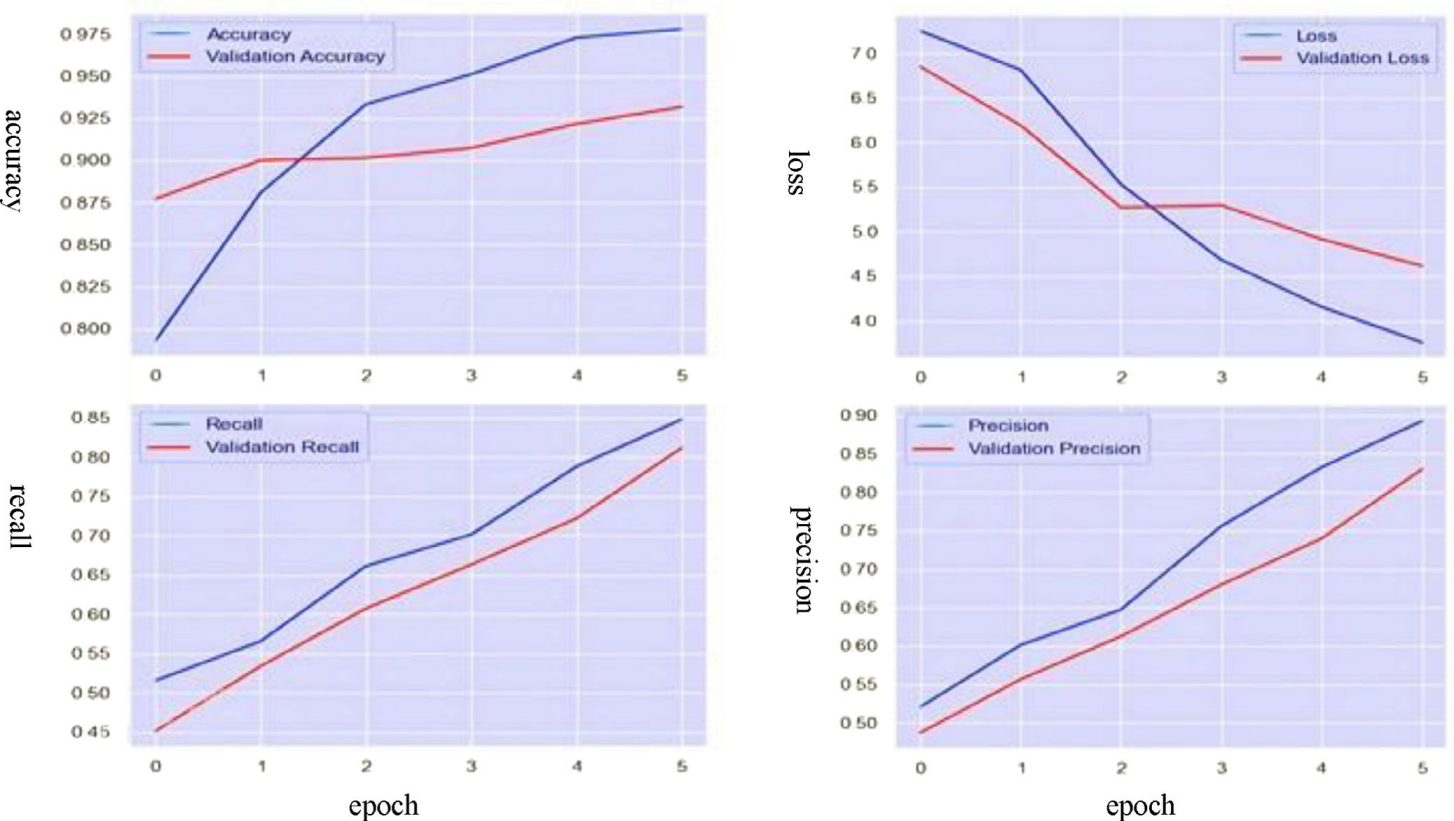
Training and validation curves of the **LSTM model**.

The LSTM model was trained for data classification over five epochs. Figure 11 depicts the training and validation curves for precision, accuracy, recall and loss. Notably, the accuracy curves for both training and validation show minimal divergence, indicating that overfitting was not a significant concern. The model achieved a maximum precision of 0.90, accuracy of 0.97 and recall of 0.85, with a final validation loss of 0.75. The LSTM approach demonstrated a consistent increase in accuracy, precision, and recall throughout training and validation. Furthermore, the final loss values in both phases displayed minimal divergence, suggesting optimal generalisation. Based on curve analysis, the LSTM model successfully mitigated overfitting and proved suitable for this task.

### 4.3 Confusion Matrix

A confusion matrix is a valuable tool for evaluating the performance of classification models. It is typically presented as a two‐dimensional table, where the rows represent the actual class labels and the columns represent the predicted labels. This matrix provides insights into classification outcomes, including correctly classified instances and cases of misclassification (Tables 4, 5, 6, 7). The XGBoost model demonstrates a slight performance improvement over KNN, as indicated by fewer misclassifications of misinformation. This suggests the favourable performance of XGBoost for this specific task. Notably, the LSTM approach demonstrates the lowest number of FNs compared to the other three models; however, it also exhibits a higher number of FPs.

**TABLE 4:** Confusion matrix in KNN test set.

Predicted negative	Predicted positive	
FN (4,058)	TP (61)	Actual positive
TN (701)	FP (165)	Actual negative

**TABLE 5:** Confusion matrix in XGBOOST test set.

Predicted negative	Predicted positive	
FN (3,984)	TP (135)	Actual positive
TN (592)	FP (274)	Actual negative

**TABLE 6:** Confusion matrix in CNN test set.

Predicted negative	Predicted positive	
FN (2,976)	TP (1,143)	Actual positive
TN (361)	FP (505)	Actual negative

**TABLE 7:** Confusion matrix in LSTM test set.

Predicted negative	Predicted positive	
FN (3,329)	TP (790)	Actual positive
TN (361)	FP (405)	Actual negative

The study used 5‐fold cross‐validation to assess model stability and prevent overfitting. The results demonstrate that the BERT model is the most robust and reliable classifier, distinguished by a negligible standard deviation (σ ≈ 0.008) in its mean performance (e.g., F1‐score). This low variance confirms that BERT's performance is highly stable across different data subsets, indicating it has successfully learnt generalisable semantic patterns rather than dataset‐specific noise. This statistical consistency highlights BERT's strength and ensures the replicability and long‐term effectiveness of the misinformation detection system for Persian COVID‐19 content on Instagram, a key advantage over traditional models with greater fluctuations. Table 8 presents the 5‐fold cross‐validation results for the models.

**TABLE 8:** 5‐fold cross‐validation on the models.

Model	F1‐score	Standard deviation (σ)
BERT (fine‐tuning)	0.915	0.008
LSTM	0.831	0.015
CNN	0.810	0.018
XGBOOST	0.852	0.012
KNN	0.785	0.022

## 5 Conclusion

This study investigated the effectiveness of XGBoost, KNN, CNN, LSTM and BERT algorithms for classifying Persian misinformation on Instagram. To prevent overfitting, training and validation curves were analysed. Additionally, confusion matrices were generated and assessed to further evaluate model performance. The results showed that the LSTM model achieved the highest classification performance among all tested architectures, reporting an F1‐score of 0.85, the best balance between precision (0.91) and recall (0.85) for this Persian social media dataset. The strong performance metrics, particularly the F1‐score, are corroborated by the training and validation curves, further confirming the model's effectiveness and generalisation ability for this high‐stakes domain.

These findings establish the first comprehensive performance benchmark for misinformation detection on Persian Instagram content. Exciting future research directions, including the development of hybrid methodologies, cross‐lingual approaches and multi‐class classification, are detailed in the Future Work section to build upon this foundational work.

## 6 Future Work

To expand upon the current research, the following future directions are suggested, prioritized for clarity and technical coherence:

1. **Prioritised recall enhancement (addressing high‐stakes FNs)**

The most critical next step is to improve the model's recall and minimise the FN rate to create a system more suitable for public health moderation. This is essential given the high‐stakes nature of misinformation detection. This enhancement can be achieved through:

- **Threshold optimisation**: Adjusting the classification threshold to prioritise the correct identification of the positive class (misinformation), even at the expense of a slight decrease in precision.
- **Feature integration**: Integrating auxiliary information such as user credibility, propagation network metrics or additional linguistic features to better detect high‐risk content.

2. **Future hybrid approaches and model integration**

Following the establishment of this performance benchmark, the logical next step is to explore hybrid methodologies for surpassing the current performance baseline. These methods could integrate the deep semantic understanding of modern deep learning models with the efficiency of classical models:

- **BERT‐XGBoost hybrid**: Using the Persian‐language BERT model (ParsBERT) to convert each post into a numerical vector (embedding) that contains rich semantic information. A classical model like XGBoost can then be trained for final classification and feature engineering on these numerical vectors. This approach aims to combine the high accuracy of BERT with the efficiency of XGBoost for scalable deployment.
- **KNN for misinformation variants**: Utilising KNN to identify posts highly similar to known false content, a method valuable for discovering repeated or slightly altered versions of misinformation.

3. **Domain and classification expansion**
  - The research can be further expanded by focusing on broader applicability and granularity:
  - **Cross‐lingual approaches**: Proposing a method for detecting social media misinformation based on bilingual and cross‐lingual approaches to leverage large, labelled English datasets and transfer knowledge to the Persian domain.
  - **Multi‐class classification**: Proposing a method for detecting social media misinformation based on multi‐class classification to determine the degree of falsehood, categorised as ‘misinformation,’ ‘accurate information’ and ‘uncertain.’ Assigning a confidence level to each classification could provide a more nuanced understanding of the misinformation landscape on social media.

## 7 Limitations

The study is limited by its focus on a single social media platform and one specific topic, which restricts the generalisability of the findings. Moreover, the dataset, drawn from a specific time window, may not fully represent the diversity of Persian‐language content on Instagram or the broader scope of misinformation. A further limitation lies in the performance metrics, specifically the recall score (0.85). For a high‐stakes public health application, this recall indicates a higher‐than‐ideal rate of FNs, meaning some harmful misinformation is missed. While the F1‐score provides a strong baseline, future work must prioritise the reduction of FNs. Additionally, the research involved lengthy preprocessing stages, which contributed to a higher computational cost.

## Author Contributions

**Mohammad Javad Shayegan**: conceptualisation, investigation, methodology, supervision, validation, writing – original draft, writing – review and editing. **Elahe Moradi**: data curation, methodology, visualisation, writing – original draft, writing – review and editing. **Mo Saraee**: investigation, validation, writing – review and editing.

## Funding

The authors have nothing to report.

## Conflicts of Interest

The authors declare no conflicts of interest.

## Data Availability

Data is available on request from the authors.
